# 3D Printing and Surface Engineering of Ti6Al4V Scaffolds for Enhanced Osseointegration in an In Vitro Study

**DOI:** 10.3390/biomimetics9070423

**Published:** 2024-07-10

**Authors:** Changyu Ma, Natan Roberto de Barros, Tianqi Zheng, Alejandro Gomez, Marshall Doyle, Jianhao Zhu, Himansu Sekhar Nanda, Xiaochun Li, Ali Khademhosseini, Bingbing Li

**Affiliations:** 1 Autonomy Research Center for STEAHM, California State University Northridge, Northridge, CA 91324, USA; 2 Terasaki Institute for Biomedical Innovation, Los Angeles, CA 90024, USA; 3 Department of Mechanical and Aerospace Engineering, University of California Los Angeles, Los Angeles, CA 90095, USA; 4 Discipline of Mechanical Engineering, Indian Institute of Information Technology, Design and Manufacturing, Jabalpur 482005, India

**Keywords:** metal additive manufacturing, titanium scaffolds, MG-63 cell, dopamine, osseointegration

## Abstract

Ti6Al4V superalloy is recognized as a good candidate for bone implants owing to its biocompatibility, corrosion resistance, and high strength-to-weight ratio. While dense metal implants are associated with stress shielding issues due to the difference in densities, stiffness, and modulus of elasticity compared to bone tissues, the surface of the implant/scaffold should mimic the properties of the bone of interest to assure a good integration with a strong interface. In this study, we investigated the additive manufacturing of porous Ti6Al4V scaffolds and coating modification for enhanced osteoconduction using osteoblast cells. The results showed the successful fabrication of porous Ti6Al4V scaffolds with adequate strength. Additionally, the surface treatment with NaOH and Dopamine Hydrochloride (DOPA) promoted the formation of Dopamine Hydrochloride (DOPA) coating with an optimized coating process, providing an environment that supports higher cell viability and growth compared to the uncoated Ti6Al4V scaffolds, as demonstrated by the higher proliferation ratios observed from day 1 to day 29. These findings bring valuable insights into the surface modification of 3D-printed scaffolds for improved osteoconduction through the coating process in solutions.

## 1. Introduction

Titanium alloy bone implants have been given high expectations in healing large-scale bone defects, attributed to their excellent biocompatibility, decent corrosion resistance, and high ratio strength [1]. With the increase in the median age of the population, bone disorders are becoming a significant concern, and bone implants are considered one feasible method to repair large bone defects with the difficulties in healing large-scale bone defects by the body [2,3]. Bone, an inhomogeneous and highly vascularized tissue, consists of the inner cancellous and outer cortical bone; the density of porous bone increases from the inner cancellous bone to the outer cortical bone. The implementation of bone implants not only supports bone tissue but also induces new blood vessel formation and benefits patients’ recovery from large bone defects [4,5]. Dense metal implants are associated with stress shielding issues due to the difference in densities, stiffness, and modulus of elasticity compared to bone tissues. A scaffold with controllable pore size is promising for reduced stress masking and improved bone formation and integration [6,7]. The parameters of the scaffold, including porosity, pore size and shape, and pore distribution, can affect bone ingrowth, while conventional methods, such as space-holder, fiber sintering, and freeze casting, are not controllable in both macro shape and micropore [8], making it of importance to develop a new fabrication route for porous scaffolds.

Additive manufacturing (AM) provides one pathway to produce porous scaffolds owing to its ability of layer-by-layer processing with micron-sized powder feedstock under an inert gas atmosphere [9]. Laser powder bed fusion (L-PBF) is an effective way to fabricate customized porous scaffolds designed by parametric porous units in the computer-aid design (CAD) software or retrieved by the Micro-Computed Tomography (CT) image [10]. L-PBF is feasible for various biocompatible materials [11,12] and porous Ti6Al4V scaffolds represent a research hot spot in the past few years. Past studies mainly focus on the design of scaffolds for decent strength. Kelly et al. [13] reported on the properties of L-PBF fabricated titanium scaffolds with a gyroid-sheet structure under different processing parameters, and their results showed conventional post-processing approaches used in solid samples do not translate to scaffolds, and more attention to the design of scaffold should be paid for improved mechanical properties. Li et al. [14] explored the relationships between pore size, shape, and mechanical properties of different additively manufactured porous titanium scaffolds while examining their viability for cell ingrowth, the results showed honeycom2 with a pore size of 300 um has not only the highest comprehensive strength, but also the highest amount of cell growth compared to the rest of scaffolds. A similar result was found in other works [15,16], where higher porosity leads to decreased tensile and compressive strength of scaffolds. Although the preferred pore size of AM-printed Ti6Al4V scaffold is still unclear [17,18], it draws much attention to the design [19] compared to surface modification of scaffolds. The uncoated titanium implants with low bioactivity can lead to loosening, or rejection coupled with bacterial colonization [20], and insufficient osseointegration in large bone defects of the skull [21], thus, coating is still critical to improve osteoconductivity and enhance osseointegration in bone implants [22]. Past research showed that plasma spray technology [23], plasma immersion ion implantation [24], plasma immersion ion implantation and deposition [25], and physical vapor deposition [26] are helpful in forming a biocompatible coating on bone implants; however, it is still challenging to produce uniform coating on L-PBF-fabricated Ti6Al4V scaffold with micron-sized pores. Thus, more efforts should be spent on coating engineering of L-PBF-produced Ti6Al4V scaffolds.

In this paper, we investigated the additive manufacturing of porous Ti6Al4V scaffolds and coating modification for enhanced osteoconduction using osteoblast cells (MG-63), a cell line derived from human osteosarcoma, that exhibits key osteoblastic properties such as high proliferation and responsiveness to osteogenic stimuli, making it ideal for bone tissue engineering research. The 3D-printed scaffolds were coated for in vitro study for a time period of up to 29 days. The results successfully showed the fabrication of Ti6Al4V scaffolds with adequate strength and surface properties suitable for good cell–material interaction, the Ti6Al4V scaffolds coated with DOPA, NaOH, or a combination of NaOH and DOPA, provide an environment that supports higher cell viability and growth compared to the uncoated Ti6Al4V scaffolds.

## 2. Materials and Methods

In this study, fabrication of porous Ti6Al4V scaffolds and coating modification for enhanced osteoconduction were conducted using osteoblast cells (MG-63) for up to 29 days.

### 2.1. Powder Material

**Figure 1:**
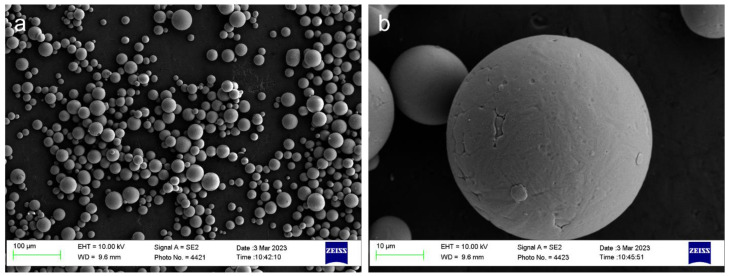
Ti6Al4V powder feedstock: (**a**) low magnification, (**b**) high magnification.

Gas-atomized spherical Ti6Al4V powder (composition as shown in Table 1) was utilized for laser powder bed fusion additive manufacturing. The particles range in size from 15 to 45 μm, where high sphericity of powder was observed through scanning electron microscopy as shown in Figure 1, with the typical satellite powder surface produced by gas atomization.

### 2.2. Design and Fabrication of Ti Scaffolds

**Figure 2:**
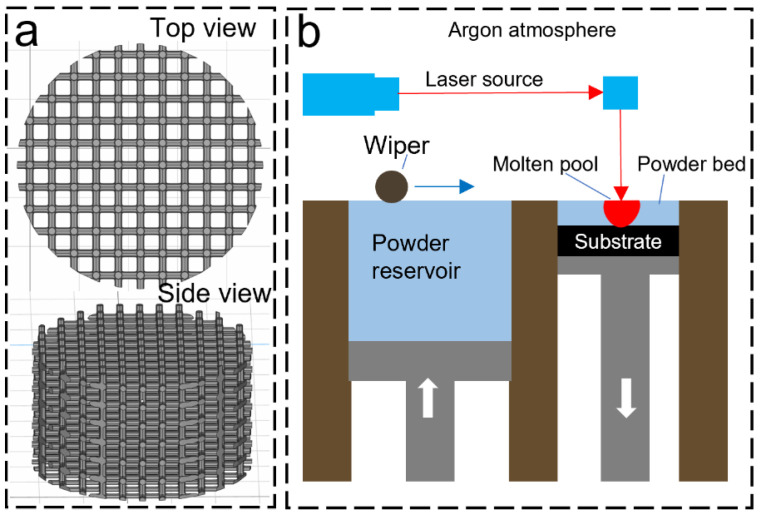
LPBF process: (**a**) CAD model of the scaffold, (**b**) LPBF process.

Ti64 scaffolds were designed using Rhino 5.0 software and fabricated on the Renishaw AM400 laser powder bed fusion (LPBF) system under Argon gas atmosphere (oxygen < 1000 ppm). According to a previous study [27], a pore size of 300 μm and a square pore shape was selected to enhance both mechanical properties and cell growth as suggested by Bidan et al. [28]. Figure 2 illustrates the design of the scaffold (5 mm in diameter, height of 5 mm) and the schematic of the LPBF process. In the LPBF process, a layer of metal powder is spread evenly on a Ti64 build plate. Then, a 200 W laser is used to selectively melt the metal powder in a predetermined pattern according to a 3D model. The laser beam is controlled by a computer, which uses a CAD (computer-aided design) file to determine the exact shape and size of each layer. The laser exposure time and point distance were set as 80 μs and 70 μm, respectively. Once the laser has melted the metal powder, the build plate is lowered by 30 μm, and another layer of metal powder is applied on top of the previously melted layer. The process is repeated until the final object is formed. During the cooling process, the melted metal solidifies and fuses with the previously melted layers, creating a strong, durable object with high accuracy and precision.

### 2.3. Finite Element Analysis

**Figure 3:**
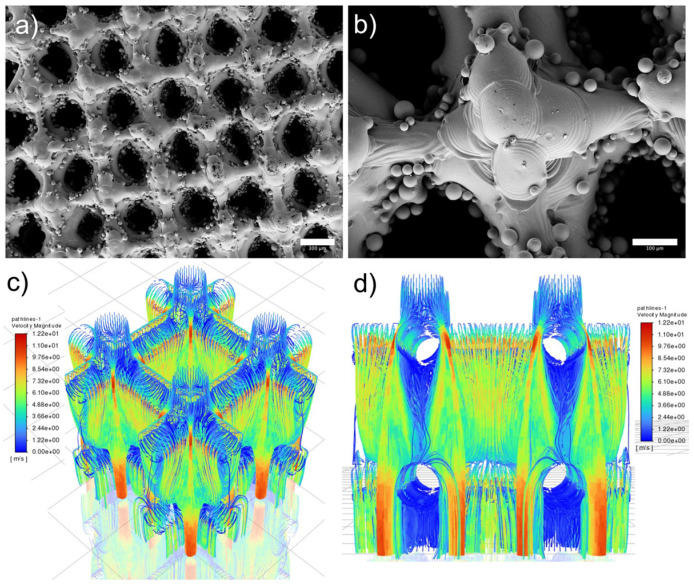
As-printed scaffold and simulation results: (**a**) top view, scale bar is 300 µm, (**b**) top view to show surface morphology, scale bar is 100 µm, (**c**) isometric view of 1000 µm unit cell with excellent liquid flow simulated through FEA and (**d**) side view of 500 µm unit cell.

The comprehensive strength and fluid permeability of scaffolds were simulated by Ansys (2021R1) software. The computational fluid dynamics (CFD) for the bone scaffold was performed through Ansys Fluent Meshing using water as a working fluid at an inlet velocity of 5 m/s. The geometry was remade in SolidWorks as a solid object, then imported and adjusted for meshing within the Ansys SpaceClaim. The model is approximated by using one unit cell since the permeability and flow pathlines are the primary concerns within this structure. Two-unit cells with dimensions of 500 and 1000 microns were utilized for the simulation of liquid flow. The 1000-micron unit cell shown in Figure 3c not only includes the 0.5 mm by 0.5 mm center unit cell but also 0.25 mm of the adjacent cells, which further increases the accuracy of the results. As shown in Figure 3d, most of the flow in a 500-micron unit cell is found to be within the center of the pathlines, with small eddies and turbulence near the surfaces. This is to be expected. The relation between the fluid flow and osseointegration is that we can approximate the osseointegration to operate as a viscous fluid, whose pathlines and relative pressures may be approximated as any other non-compressible working fluid.

### 2.4. Surface Engineering of Ti64-Scaffolds

Control Ti64 scaffolds were manufactured using the laser powder bed fusion (LPBF) system Renishaw AM400 (Renishaw plc, Wotton-under-Edge, UK). Ti64 scaffolds were cleaned using 1 h of sonication using an ultrasonicated water bath (Emerson CPX1800H, St. Louis, MO, USA) and rinsed with DI water and absolute (100%) Et-OH to make sure all the loosely bound metal powders were removed from the samples. Cleaned Ti64 scaffolds were dried using Kimwipes wipers and kept in separate glass vials/beakers. The scaffolds were dried for a few minutes in an incubator to make sure the surface of the scaffolds was dry enough for further treatment. The dried scaffolds were boiled with NaOH (5M) at 80–90 °C for 72 h in a reagent bottle. The boiled scaffolds were recovered and washed with DI water and dried. 200 mL of Dopamine Hydrochloride (DOPA) (1 mg/mL) dissolved in Tris HCl (pH 8.5) was prepared in a 500 mL flask. The dried scaffolds were immersed inside the dopamine solution and surface coating was carried out for 24 h at room temperature under stirring conditions. The coated scaffolds were recovered, washed, and dried for SEM observation. For direct dopamine coating, the scaffolds were added directly into the prepared dopamine solution, and the surface coating was carried out with the procedure as described. Ti64 scaffolds treated with NaOH, DOPA, or a combination of NaOH and DOPA were observed for their surface morphology using a scanning electron microscope (SEM, ZEISS Supra 40VP, Pleasanton, CA, USA). Images of the scaffolds were captured at an acceleration voltage of 10 kV.

### 2.5. Compression Test

The compressive mechanical strength of the original and coated scaffolds was evaluated through the Instron 5966 universal testing system (Instron, High Wycombe, UK). Compression tests were conducted at room temperature with a load cell of 10 KN, the preloading force was set to be 50 N, with a loading speed of 8.47 µm/s.

### 2.6. In Vitro Study

*In vitro cytocompatibility and proliferation:* This study employs in vitro methods to assess the cytocompatibility and proliferation of human osteoblast cells on 3D-printed Ti6Al4V porous scaffolds. We conducted these experiments to evaluate how well the cells adhere to, grow on, and interact with the scaffold surfaces. By using in vitro techniques, we can precisely monitor cellular behavior and scaffold performance, which are crucial for determining the potential for enhanced osseointegration. The 3D-printed scaffolds with different coatings were seeded with an osteoblast cell line with fibroblast morphology (MG-63, CRL-1427™-ATCC). A total of 5 × 10^5^ cells were seeded per scaffold on low attachment 24-well plates. At specific time points after cell seeding (1, 3, 7, 14, 21, and 29 days), the scaffolds with attached cells were transferred to new wells to avoid measuring unattached cells, and metabolic activity was quantitatively assessed by the PrestoBlue cell viability reagent (Thermo Fisher Scientific, Eugene, OR, USA), and cell viability was evaluated qualitatively by a Live/dead cell imaging kit (Thermo Fisher Scientific, Eugene, OR, USA). Live/dead fluorescent images were obtained using an inverted fluorescence microscope (Zeiss Axio Observer 5 microscope, Pleasanton, CA, USA) or a confocal microscope (Zeiss LSM880, CA, USA).

*Cell attachment and morphological studies:* This study utilizes in vitro methods to investigate the attachment and morphological characteristics of human osteoblast cells on 3D-printed Ti6Al4V porous scaffolds. By examining how human osteoblast cells adhere to and spread across the scaffold surfaces, we can gain detailed insights into the biocompatibility and structural suitability of the scaffolds. In vitro analysis allows for controlled observations of cellular interactions and morphological adaptations, essential for assessing the potential of these scaffolds in enhancing osseointegration. The 3D-printed scaffolds were seeded with MG-63 cells following the same procedures for cell cytocompatibility. At 24 h after cell seeding, live/dead fluorescent images were utilized to measure the area of attached cells compared to control samples (3D-printed scaffolds without coating). For morphological studies, samples were stained for cell nuclei (DAPI-4′,6-diamidino-2-phenylindole, Sigma-Aldrich, St. Louis, MO, USA) and F-actin (Alexa Fluor 568 Phalloidin, Thermo Fisher Scientific, Eugene, OR, USA) after seeding and culturing osteoblasts on the 3D-printed scaffolds for up to 29 days. Fluorescent images were obtained using an inverted fluorescence microscope (Zeiss Axio Observer 5 microscope, Pleasanton, United States) or a confocal microscope (Zeiss LSM880, Pleasanton, CA, USA). The cell-seeded 3D-printed scaffolds were coated with Au for SEM observation.

*Protein expression analysis:* This study uses in vitro techniques to perform protein expression analysis on human osteoblast cells cultured on 3D-printed Ti6Al4V porous scaffolds. By analyzing the expression levels of specific proteins, we aim to understand the cellular responses and pathways activated in response to the scaffold surfaces. In vitro protein expression analysis provides precise and controlled insights into the molecular mechanisms underlying scaffold biocompatibility and osseointegration potential. For that, immunofluorescence assessed RUNX2 (anti-RUNX2 antibody, clone AS110, Millipore Sigma, St. Louis, MO, USA), Collagen I (anti-Collagen I antibody, ab34710, Abcam, Waltham, MA, USA), and Osteocalcin (anti-Osteocalcin antibody, clone 7D1.13, Millipore Sigma, St. Louis, MO, USA) expression of cells seeded on 3D-printed scaffolds. Primary antibodies were utilized at 1:200 dilution. Secondary antibodies: Goat anti-Rabbit IgG (H + L) Alexa Fluor 594 and Rabbit anti-Mouse IgG (H + L) Alexa Fluor 488 (Invitrogen, Carlsbad, CA, USA) were utilized at 1:200 dilution. The samples were also stained for cell nucleus and F-actin. Immunofluorescent images were obtained using an inverted fluorescence microscope or a confocal microscope. Scanning electron microscopy also assessed the cell-seeded 3D-printed scaffolds. After cell seeding, samples were evaluated on days 1, 7, and 29.

All the in vitro studies in this work were performed at the Terasaki Institute for Biomedical Innovation (TIBI), Los Angeles, CA, USA, between 2022 and 2023. Since all our in vitro studies were performed using an established human cell line (MG-63), ethical committee approval was not required under state regulations and our institution’s policies.

### 2.7. Statistical Analysis

The experiments were conducted in a minimum of three independent sessions, and the data is expressed as mean ± standard deviation. GraphPad Prism v10.2.2 software was used to perform statistical analysis. For the comparison of the means between two independent groups, a two-tailed unpaired t-test was used. To compare the means of three or more independent groups, One-way ANOVA with a Tukey multiple comparison test was utilized. Additionally, Two-way ANOVA was employed for the comparison of the means of the groups over time. A probability value (*p*) of <0.05 was considered statistically significant.

## 3. Results

### 3.1. Ti64 Scaffolds

Figure 3 illustrates the surface morphology and FEA simulation results of scaffolds. Figure 3a,b display the top view of as-printed scaffolds, where partially melted particles (up to 45 microns) were observed on the surface. The powder attached to the scaffold is caused by the partial melting during the LPBF printing, the mean pore size of as-printed scaffolds measured is 279.3 μm. In addition, our previous study demonstrated that as-printed scaffold has an Ra of 3.35 μm which benefits cell growth [27]. It is worth noting that the measurement is taken in the smooth areas without partially melted powders, surface coating is a promising avenue to reduce the surface roughness caused by these powders and improve the cell viability. Figure 3c,d show the liquid flow with the highest velocity magnitude of 12.2 near the surface area, with reduced velocity magnitude observed in the center of the scaffold.

### 3.2. Scaffold Engineering

**Figure 4:**
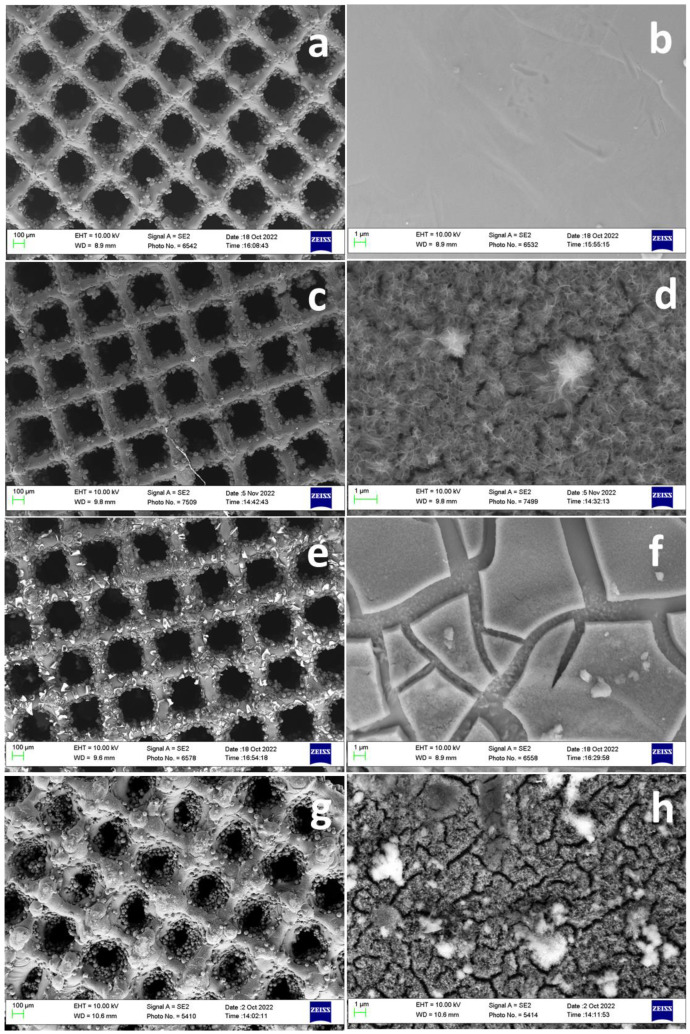
Surface morphology of surface treated scaffolds. Control Ti64 (**a**,**b**), Ti64 scaffold boiled with 5M NaOH (72 h) at 90 °C (**c**,**d**), Ti64 scaffolds treated directly with DOPA (24 h) at RT (**e**,**f**), Ti64 scaffold boiled with 5M NaOH (72 h) at 90 °C and further treated with DOPA (24 h) (**g**,**h**).

Figure 4 shows the morphology of modified surfaces. Figure 4a,b show the surface without any modifications which represents a smooth surface. Figure 4c–h represents the surfaces of Ti64 scaffolds after various modifications. The high magnification images (Figure 4d,f,h) of modified surfaces showed a distinct morphological appearance compared to the control Ti64. NaOH-boiled Ti64 scaffolds exhibited nanoscale porous network structure (Figure 4d). The porous structure is predominantly related to the dissolution of the oxide layer caused by NaOH treatment. The porous network structure could disappear after DOPA grafting (Figure 4h). This indicated the adhesive layer could tightly bound to the nanoscale surface via chemical and mechanical interlocking which may further facilitate the controlled release of DOPA from the scaffold surface. DOPA can promote osteogenesis and prevent osteoclastogenesis [29]. Thus, its controlled release from the surface of Ti64 scaffolds may increase the resistance of Ti64 scaffolds to bone resorption and improve the bone regeneration process. However, the scaffolds directly treated with DOPA showed the loosely bonded adhesive PDA layer on the surface which is distributed with a high degree of non-uniformity like the peeled layers with cracks which may hamper the durability of the coatings. Overall, different kinds of chemical treatment over Ti64 scaffolds entail different surface morphology as well as a surface quality which may further influence cell behavior.

### 3.3. Compression Test

To prove the concept, the compressive strength of as-printed and coated scaffolds was revealed to show that coated scaffolds possess excellent strength as bone implants shown in Table 2. The as-printed scaffold with a compressive strength of 149.7 ± 4.9 MPa, and the compressive strength for NaOH, DOPA, and NaOH plus DOPA-treated scaffolds are 150.5 ± 6.7, 155.8 ± 7.1, and 141.8 ± 4.6 MPa, respectively. It demonstrates the coating effects on the mechanical properties of the scaffold are negligible, considering the reasonable deviation between the tests.

### 3.4. In Vitro Studies with Osteoblasts

The present study investigates the in vitro performance of 3D printed porous titanium scaffolds subjected to four distinct surface treatments: (i) no treatment as a control, (ii) sodium hydroxide (NaOH) treatment, (iii) dopamine treatment, and (iv) a combination of sodium hydroxide and dopamine treatment. The evaluation of human osteoblasts’ response to these modified scaffolds will shed light on the effects of surface treatment on cell adhesion, proliferation, and differentiation, ultimately guiding the design and optimization of advanced bone tissue engineering implants with enhanced osseointegration and bone regeneration potential.

**Figure 5:**
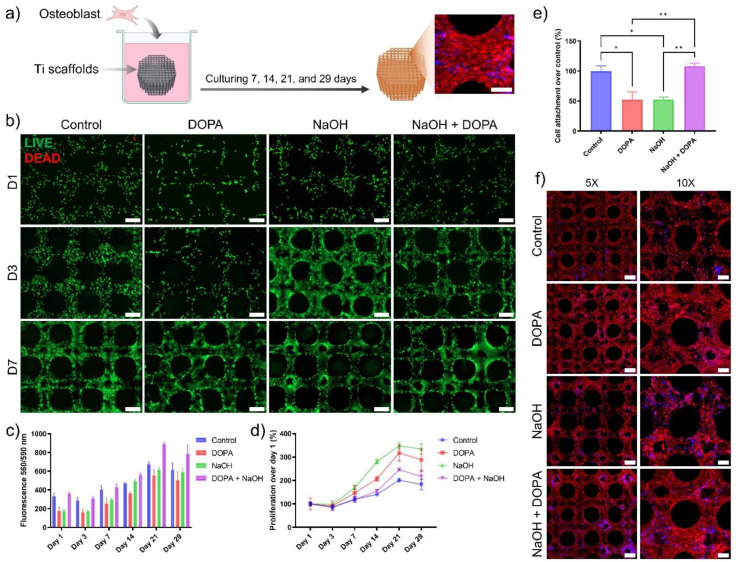
In vitro studies of osteoblast adhesion, viability, and proliferation on coated 3D-printed porous titanium scaffold: (**a**) Schematic of osteoblast seeding on 3D-printed porous titanium scaffolds and culture for up to 29 days. (**b**) Representative fluorescence images of live/dead staining to assess cell adhesion and viability. (**c**) Fluorescence levels for osteoblasts cultured on titanium scaffolds to assess metabolic activity. (**d**) Proliferation curves of osteoblasts cultured on the porous titanium scaffolds. (**e**) Quantification of cell adhesion on titanium scaffolds 24 h after cell seeding (* *p* < 0.05, ** *p* < 0.01). (**f**) Representative fluorescence images of osteoblasts stained for cell nucleus and acting after 29 days of culture on titanium scaffolds. Scale bar is 200 µm.

The in vitro cytocompatibility and proliferation study evaluated the response of 3D-printed porous titanium scaffolds treated with different surface coatings using an osteoblast cell line. Briefly, 3D-printed scaffolds with different coatings were seeded with osteoblast cells at 5 × 10^5^ cells per scaffold on low attachment 24-well plates, and cell behavior was assessed at specific time points (Figure 5a). Live/dead staining and metabolic activity assessments were conducted at specific time points following cell seeding to quantify cell viability and proliferation. Remarkably, all groups demonstrated high cell viability throughout the study, with minimal cell death observed in the live/dead stains at all time points (Figure 5b). The metabolic activity analysis revealed reduced fluorescence levels in the NaOH and DOPA-treated groups on day one (Figure 5c). However, this initial decrease in metabolic activity did not compromise cell proliferation (Figure 5d).

Further investigation revealed that the reduced fluorescence levels for the metabolic activity of NaOH and DOPA-treated groups were attributed to a lower number of cells attached to the scaffolds during the first 24 h (Figure 5b,e). Despite this, all treated groups exhibited enhanced cell proliferation compared to the control group, with the NaOH and DOPA-treated groups showing the highest proliferation rates up to 21 days (Figure 5d). After 21 days, the surface of the porous scaffolds in all groups was covered entirely with cells, indicating successful cell adhesion and growth on the scaffolds.

The cell attachment study conducted during the first 24 h after cell seeding measured the area of fluorescence signal from the live/dead stains to assess cell attachment. The NaOH and DOPA-treated groups exhibited a reduced percentage of attached cells, approximately 50% less compared to the control and NaOH + DOPA-treated groups (Figure 5e). Subsequently, morphological studies were performed using representative stains for DAPI and phalloidin to visualize cell nuclei and actin, respectively, after 29 days of culturing osteoblast-seeded scaffolds. The control and NaOH-treated groups displayed similar morphology with moderate surface coverage (Figure 5f). In contrast, the DOPA and NaOH + DOPA-treated groups showed significantly improved surface coverage, with a notable increase in the signal for actin, indicative of enhanced cell adhesion and cytoskeletal organization.

**Figure 6:**
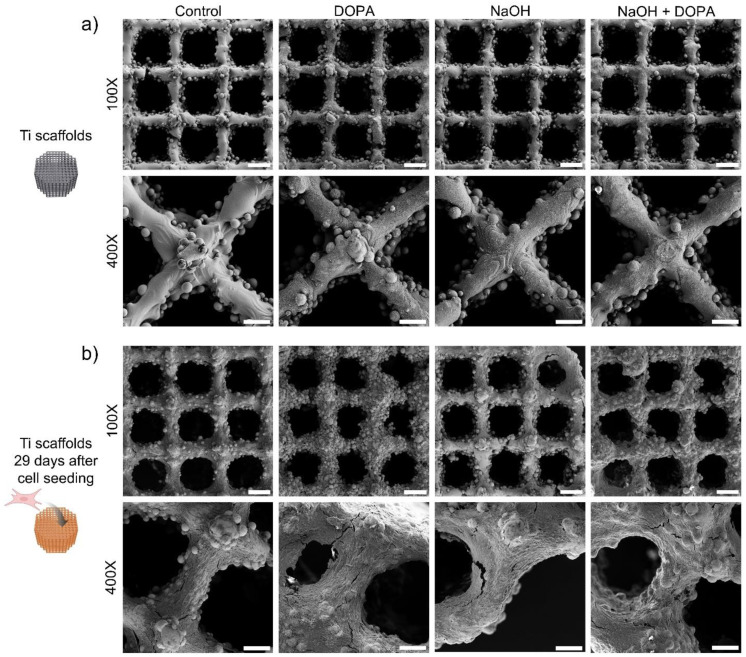
Surface morphology of coated 3D-printed porous titanium scaffold with osteoblasts seeded and cultured for up to 29 days. Representative SEM images of 3D-printed porous titanium scaffolds: (**a**) before cell seeding and (**b**) after culturing osteoblasts for 29 days. Scale bars are 300 µm (100X) and 80 µm (400X).

These observations were further corroborated by SEM images taken after 29 days of osteoblast culturing on the porous scaffolds. Before cell seeding, the porous scaffolds exhibited a surface with melted titanium comprising the lattice structure, along with partially melted titanium particles contributing to the scaffold’s roughness (Figure 6a). Importantly, all the treated groups displayed microscopic differences compared to the control group, indicating the effectiveness of the surface treatments in altering scaffold characteristics to facilitate cell interaction.

Notably, after 29 days of osteoblast culture on the porous scaffolds, all tested groups exhibited complete coverage with cells, signifying successful cell adhesion and proliferation. Among them, the DOPA and NaOH + DOPA-treated groups demonstrated the most improved surface coverage with osteoblasts (Figure 6b), reaffirming the favorable influence of these surface treatments on promoting cell attachment and growth.

**Figure 7:**
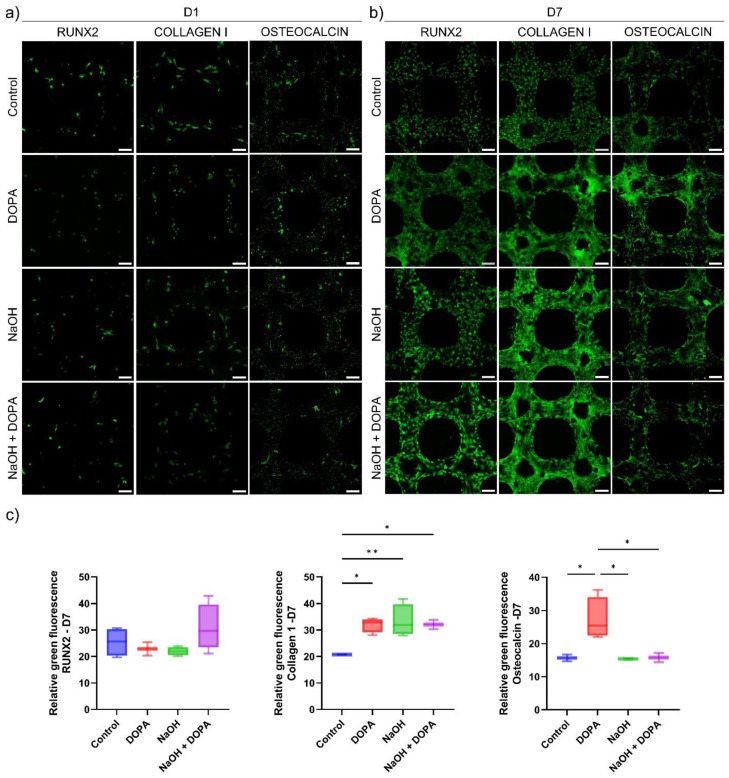
Protein expression studies of osteoblast cultured on coated 3D-printed porous titanium scaffolds. Representative fluorescence images for RUNX-2, Collagen 1, and Osteocalcin expression in osteoblasts cultured on the porous titanium scaffolds for (**a**) one day and (**b**) seven days. Scale bar is 100 µm. (**c**) Relative fluorescence intensity for protein expression at seven days of osteoblast culture on the porous titanium scaffolds. (* *p* < 0.05, ** *p* < 0.01).

The protein expression analysis through immunofluorescence provided valuable insights into the osteoblast response to the 3D-printed porous titanium scaffolds. Specifically, the expression of key osteogenic markers, including RUNX-2, Collagen 1, and Osteocalcin, was evaluated at day seven of culture for all treatment groups (Control, NaOH, DOPA, and NaOH + DOPA) (Figure 7).

The results revealed that the expression of RUNX-2, a critical transcription factor involved in osteoblast differentiation, showed a notable increase in the NaOH + DOPA-coated group compared to the other treatment groups (Control, NaOH, and DOPA). Although this difference in RUNX-2 expression was not statistically significant, it indicated a potential trend of enhanced osteoblastic differentiation in the NaOH + DOPA-treated group (Figure 7c).

On the other hand, the expression of Collagen 1, a major component of the extracellular matrix in bone tissue, exhibited significant upregulation in all the treatment groups (NaOH, DOPA, and NaOH + DOPA) compared to the Control group (Figure 7c). This enhanced Collagen 1 expression in the treated groups suggested improved extracellular matrix deposition and potential for enhanced bone matrix formation.

Furthermore, the expression of Osteocalcin, a late-stage marker of osteoblast differentiation, showed a significant increase specifically in the DOPA-coated group compared to the other treatment groups and the Control (Figure 7c). This indicated that DOPA surface treatment might particularly promote osteoblastic maturation and mineralization processes.

The combined results from the protein expression analysis through immunofluorescence provided valuable molecular insights into the osteogenic potential of the 3D-printed porous titanium scaffolds with different surface treatments. The upregulation of Collagen 1 in all treated groups suggested the scaffolds’ capacity to support extracellular matrix production, while the significant increase in Osteocalcin expression in the DOPA-coated group indicated its potential for enhancing late-stage osteoblastic differentiation. Moreover, the observed trend of increased RUNX-2 expression in the NaOH + DOPA-coated group highlighted a potential synergistic effect of combined surface treatments on osteoblast differentiation. Collectively, these findings contribute to a comprehensive understanding of the cellular responses to surface-treated scaffolds and provide valuable information for the development of optimized bone tissue engineering implants with enhanced bone regenerative potential.

## 4. Discussion

In recent years, 3D printing has emerged as a revolutionary technique for fabricating complex structures with tailored porosity, leading to the development of porous titanium scaffolds for bone tissue engineering applications [30,31]. These scaffolds offer great potential in regenerative medicine because they provide mechanical support, facilitate cell adhesion, and permit nutrient diffusion [32]. However, the surface characteristics of these scaffolds play a crucial role in determining their biocompatibility and osteogenic properties. The comprehensive investigation of 3D-printed porous titanium scaffolds with different surface treatments in this study has yielded crucial insights into their potential for bone regeneration applications. Various chemical methods have been used to modify the surface of scaffolds as these techniques are minimally cytotoxic and biologically safe. Surface engineering of scaffolds provides improved surface hydrophilicity and enhanced cell-materials interactions. This may positively modulate the pro-angiogenic, antibacterial, and anti-biofilm properties of the scaffolds [33]. In this study, the chemical method of surface treatment was carried out using NaOH, Dopamine (DOPA), and a combination thereof. NaOH treatment over the implants has widely been adapted to increase the surface roughness. For example, the treatment of Ti implants with NaOH produces a fine network structure of nanometer scale on the surface of the Ti metal [34]. These nanoscale features have been shown to enhance the apatite formation over the surface and are well-proven to improve the osseointegration potential of Ti-based implants. Similarly, polydopamine (PDA) coatings have been widely studied as a method to improve the bioactivity of the surface of metals [35,36]. PDA is known to form in an oxidative reaction between dopamine and oxygen in alkaline conditions and can adhere to metal surfaces in a simple dip coating technique. PDA is well known as a bioadhesive which may provide several positive attributes to an implant surface such as antibacterial, antifungal, antioxidant, photothermal, reactive oxygen species (ROS) scavenging, and separation of metal cations. Its low cost as well as easy availability has attracted much attention for surface modification.

The results from cell viability and proliferation assays demonstrated excellent cytocompatibility and cell growth on all treated scaffolds, indicating their biocompatibility and capacity to support osteoblast proliferation, critical for bone tissue regeneration. Similar results regarding cell adhesion and proliferation on 3D-printed porous titanium alloy scaffold were broadly described [37,38,39].

The morphological studies further corroborated these findings, revealing enhanced cell attachment and coverage, especially in the DOPA and NaOH + DOPA-treated groups. Such enhanced cell attachment is vital for facilitating the formation of a stable and functional bone-tissue interface. The functionalization of 3D-printed porous titanium scaffolds has been demonstrated with a variety of materials, including hydroxyapatite, calcium phosphate, bioactive glass, collagen, and chitosan [40,41,42,43,44]. Although different coating methods have been developed and shown to improve cell adhesion and proliferation, the expression of early and late osteodifferentiation markers is of the most importance in defining a promising material for bone regeneration.

The protein expression analysis through immunofluorescence provided valuable molecular evidence to support the positive impact of surface treatments on osteogenic processes. The significant upregulation of Collagen 1 in all treated groups indicated the scaffolds’ ability to promote extracellular matrix deposition, crucial for providing the necessary structural support during bone regeneration. Moreover, the remarkable increase in Osteocalcin expression specifically in the DOPA-coated group signified the potential of this treatment to induce osteoblastic maturation and mineralization processes, crucial for the development of fully functional bone tissue. The observed trend of increased RUNX-2 expression in the NaOH + DOPA-treated group further underscored the potential synergistic effect of combined surface treatments on osteoblast differentiation, suggesting a possible strategy to enhance osteogenic outcomes in bone regeneration applications.

Sheng et al. demonstrated improved osteogenic differentiation in a 3D-printed titanium alloy microporous interface coated with collagen functionalized with calcium phosphate [43]. Additionally, cells cultivated on the collagen-functionalized scaffolds presented a significant increase in RNA expression of *ALP*, *BMP-2*, *OCN*, and *MMP-2*, underscoring the osteogenic potential of coated titanium scaffolds for bone tissue engineering.

Therefore, the comprehensive investigation of 3D-printed porous titanium scaffolds with various surface treatments has provided significant insights into their potential for bone regeneration applications. The study’s findings, encompassing cell viability, proliferation assays, and morphological studies, affirm the biocompatibility and osteoconductivity of treated scaffolds. The superior cell attachment and coverage, particularly in DOPA and NaOH + DOPA-treated groups, highlight the importance of surface functionalization in promoting a stable bone-tissue interface. Furthermore, the upregulation of critical osteogenic markers, such as Collagen 1, Osteocalcin, and RUNX-2, underscores the efficacy of these treatments in enhancing osteoblast differentiation and maturation. The promising results from protein expression analysis and corroborative studies with coatings on titanium porous scaffolds further reinforce the osteogenic potential of these scaffolds. Therefore, the strategic combination of advanced surface treatments holds considerable promise for advancing bone tissue engineering and achieving successful bone regeneration.

## 5. Conclusions

The combination of positive outcomes from cell viability, proliferation, morphology, and protein expression analyses highlights the promising potential of the surface-treated 3D-printed porous titanium scaffolds for bone regeneration. The ability to promote cell adhesion, proliferation, extracellular matrix production, and osteoblastic differentiation makes these scaffolds highly attractive candidates for use in advanced bone tissue engineering implants. These findings pave the way for further advancements in biomaterial design, leading to the development of innovative and effective strategies for bone regeneration, ultimately improving the quality of life for patients suffering from bone injuries or defects. As the field of regenerative medicine continues to evolve, the knowledge gained from this study holds great promise for the translation of these scaffold technologies from the laboratory to clinical applications, revolutionizing the future of bone tissue engineering and regenerative medicine.

## Figures and Tables

**Table 1:** Compositions of Ti6Al4V.

Elements	Ti	Al	V	Fe	O	C	N	H	Y	Residuals
Mass (%)	Bal.	5.50~6.50	3.50~4.50	≤0.25	≤0.13	≤0.08	≤0.05	≤0.012	≤0.005	≤0.4

**Table 2:** Compressive strength of scaffolds.

Specimens	Compressive Strength (MPa)
Control	149.7 ± 4.9
Ti-NaOH	150.5 ± 6.7
Ti-DOPA	155.8 ± 7.1
Ti-NaOH + DOPA	141.8 ± 4.6

## Data Availability

Data are contained within the article.
